# Response of Resistance Exercise-Induced Muscle Protein Synthesis and Skeletal Muscle Hypertrophy Are Not Enhanced After Disuse Muscle Atrophy in Rat

**DOI:** 10.3389/fphys.2020.00469

**Published:** 2020-05-21

**Authors:** Satoru Ato, Kohei Kido, Kohei Sase, Satoshi Fujita

**Affiliations:** ^1^Graduate School of Sport and Health Science, Ritsumeikan University, Shiga, Japan; ^2^Department of Life Science and Applied Chemistry, Nagoya Institute of Technology, Nagoya, Japan; ^3^Laboratory of Sports and Exercise Medicine, Graduate School of Human and Environmental Studies, Kyoto University, Kyoto, Japan

**Keywords:** skeletal muscle, disuse atrophy, resistance training, muscle protein synthesis, mTORC1

## Abstract

Skeletal muscle disuse rapidly decreases muscle mass. Resistance training (RT) is believed as the most effective way to gain muscle mass via an increase in mTORC1 activity and muscle protein synthesis (MPS). However, it remains unclear whether muscle atrophy by disuse alters the mTORC1 activation and MPS response to an acute resistance exercise (RE) and chronic RT–mediated skeletal muscle hypertrophy. This study investigated the influence of disuse muscle atrophy on the response of mTORC1 activation and MPS to an acute RE. We also evaluated whether disuse muscle atrophy affects the response of RT-induced muscle mass gain. Thirty male Sprague-Dawley rats were randomly divided into control (CON) or hindlimb suspension (HS) groups. A 14-day HS via the tail was used as the model for gastrocnemius muscle disuse in the HS group. Unilateral lower limb muscle contraction using by percutaneous electrical stimulation was used to mimic the stimuli of RE. Ten bouts of RE were performed in 3-week as chronic RT. Our results showed that MPS and mTORC1 activity was unchanged after HS at basal state. However, the ribosomal RNA (rRNA) level was reduced in HS rats compared to that in CON rats at basal state. MPS and rRNA increased in both HS and CON rats in response to acute RE to the same extent. However, the level of mTORC1 activation in response to an acute RE was significantly higher in HS than that in the CON group at 12 h after exercise, even though no difference was observed at 3 h after exercise. The 10-bout RT significantly increased gastrocnemius muscle mass in both CON and HS rats. The response of muscle hypertrophy did not differ between the groups. Therefore, MPS in response to acute RE and muscle hypertrophy in response to chronic RT were unaltered after disuse muscle atrophy.

## Introduction

Skeletal muscle is known to have a broad range of plasticity, such as hypertrophy in response to mechanical loading [i.e., functional overload, resistance training (RT)] (Goodman et al., 2011a; Ogasawara et al., 2016a), and atrophy in response to disuse (i.e., space flight, bed rest, and inactivity; Allen et al., 1996; Glover et al., 2008; Drummond et al., 2012). The net balance of muscle protein metabolism that is regulated by protein synthesis and breakdown is one of the key determinant factors of muscle mass (Russell, 2010). Muscle protein synthesis (MPS) is believed to be regulated by translational efficiency and translational capacity. Mammalian target of rapamycin (mTOR) is believed as a key regulator of mRNA translation and cell growth (Sabatini, 2017). mTOR is known to exist as at least two molecular complexes (mTORC1 and mTORC2). In particular, activated mTORC1 phosphorylates p70S6K Thr389 and 4E-BP1 Thr37/46 in which positively regulates translation initiation (Mamane et al., 2006). Previous studies revealed that mTORC is a key regulator of muscle contraction-induced MPS and skeletal muscle hypertrophy (Goodman et al., 2011a; West et al., 2016; Ogasawara et al., 2016b; Ogasawara and Suginohara, 2018; You et al., 2018). Ribosomes play an essential role in the molecular machinery for protein synthesis and are composed of several different ribosomal RNAs (rRNAs) and ribosomal proteins (Lafontaine and Tollervey, 2001). A previous study observed that resting intracellular ribosomal content is highly correlated to the MPS (Millward et al., 1973). Moreover, recent evidence suggests that the capacity of ribosome biogenesis is relevant to the response of load-induced skeletal muscle hypertrophy (von Walden et al., 2012; Kirby et al., 2015; Figueiredo and McCarthy, 2019).

Skeletal muscle unloading, such as bed rest, cast immobilization, and space flight, markedly decrease muscle mass (Allen et al., 1996; Glover et al., 2008; Drummond et al., 2012; Fox et al., 2014; You et al., 2015; Mirzoev et al., 2016; Baehr et al., 2017). Previous studies have reported that MPS was significantly reduced, and mTORC1 activity along with ribosomal content decreased during muscle atrophy (Machida et al., 2012; Baehr et al., 2017). Thus, it is believed that reduction in MPS is a partial cause of the loss in muscle mass under muscle disuse conditions. Since acute resistance exercise (RE) stimulates mTORC1 activation, MPS gain and ribosome biogenesis and repeated RT results in muscle mass gain (Ogasawara et al., 2016b), RT is assumed as an efficient strategy to rapidly recover, and/or improve skeletal muscle mass. Although a large part of the mechanism underlying RT-induced muscle hypertrophy remain unclear, it is believed that mTORC1 activation and increase in MPS to an acute RE is important for RT-induced muscle hypertrophy (Glass, 2003; Goodman et al., 2011a; West et al., 2016; Ogasawara and Suginohara, 2018; You et al., 2018; Ogasawara et al., 2019). Interestingly, a previous murine study observed that limb immobilization sensitized MPS in response to muscle contraction (You et al., 2015). Thus, the MPS response to an acute RE may be enhanced under the conditions of disuse muscle atrophy. On the contrary, it is known that response of MPS to nutritional intake is impaired under condition of muscle atrophy (Glover et al., 2008; Drummond et al., 2012). Thus, it is remained controversial that understanding of the effect of disuse-induced muscle unloading on the response of MPS to anabolic stimuli (e.g., nutrition, muscle contraction). Furthermore, previous studies discussed that response of muscle mass gain to contraction is grater when regaining as compared to the muscle mass gain from a native state (Bruusgaard et al., 2012; Lee et al., 2018). These phenomena have observed in rodent and human and is recognized as “muscle memory” (Staron et al., 1991; Lee et al., 2018). Even though large part of mechanisms remain to be determined, unload-induced changing the response of MPS to muscle contraction might contribute as a part mechanism underlying “muscle memory.” Molecular understanding of the MPS response and muscle mass regain to RT after disuse-induced muscle atrophy may serve to provide efficient skeletal muscle recovery strategies after prolonged-bed rest or space flight. Furthermore, it would contribute to elucidate a part of the mechanisms of “muscle memory.” However, it is unclear whether the MPS response to an acute RE is altered after disuse muscle atrophy. In addition, no study has directly evaluated whether the muscle mass gain in response to RT is altered after disuse muscle atrophy.

Therefore, this study aimed to investigate the effect of disuse muscle atrophy on the muscle hypertrophy response to chronic RT. Furthermore, this study assessed the MPS response to acute RE after unloading-induced skeletal muscle atrophy.

## Materials and Methods

### Experimental Design

**FIGURE 1:**
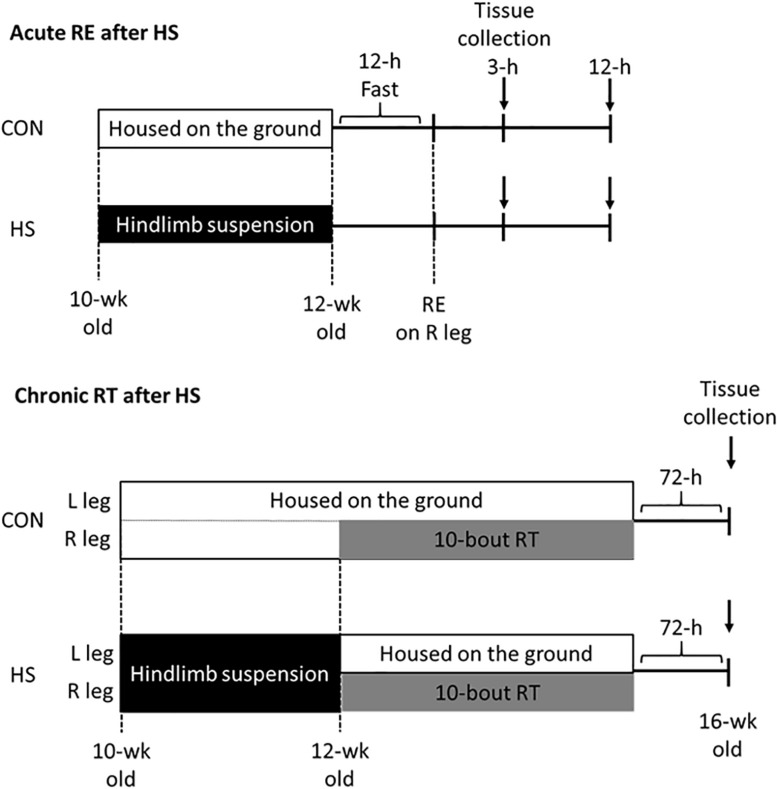
Schematic representation of hindlimb suspension and exercise training intervention. CON; control group. HS; hindlimb suspension group. RE; acute resistance exercise. RT; resistance training.

The study protocol was approved by the Ethics Committee for Animal Experiments at Ritsumeikan University, Japan (BKC2016-030). Male Sprague-Dawley rats, aged 9 weeks, were purchased from CREA Japan (Tokyo, Japan). All rats were acclimated for 1 week in an environment kept at 22–24°C with a 12 h–12 h light-dark cycle and were given *ad libitum* access to food and water. Following the acclimation, the rats were randomly divided into two groups (*n* = 15/group): the control (CON) group, and hindlimb suspension (HS) group. The HS group performed 14-day hindlimb unloading using by the tail suspension method to induce disuse muscle atrophy. The CON group was kept at the ground level during the intervention period. 14 days after HS, each group was subdivided into the acute RE and chronic RT groups. Acute RE group (*n* = 5–6/time point/group) was subjected to an overnight fast, followed by acute RE (isometric maximal contraction) using the right hindlimb muscle with the use of percutaneous electrical stimulation. Skeletal muscles were harvested at 3 h and 12 h after the exercise. Chronic RT rats (*n* = 5/group) performed RT (chronic RE) 3 times a week (i.e., Mon-Wed–Fri), for a total of 10-bouts. The rats in the HS group returned to the ground level during the chronic training intervention periods (Figure 1). Seventy-two h after the final exercise bout, skeletal muscles were obtained as samples.

Euthanasia was performed by blood removal from the heart under isoflurane anesthesia. After the blood removal, gastrocnemius and soleus muscles were removed and weighed. The sample tissue was kept at −80°C until analysis.

### Hindlimb Suspension

Hindlimb suspension was performed using a rat HS instrument (Yamashita Giken, Tokushima, Japan). In detail, the rat tail was washed with a neutral detergent to remove the sebum under isoflurane-inhaled anesthesia. Subsequently, the tail was wrapped with a urethane foam pad and held-up with a clip. It was suspended on a rail frame so that the body was inclined 30° forward and the leg did not touch the floor. The rats in the CON group were kept for a similar time period as that of the treatment of tails in the HS group, under anesthesia, as a sham operation (∼10 min).

### Resistance Exercise

Resistance Exercise was performed as previously described (Ato et al., 2016; Ato et al., 2017). Under isoflurane-inhaled anesthesia, the hair on the right hindlimb was shaved, and the area was cleaned up with alcohol wipes. The rats were then positioned with their right foot on a footplate in the prone position. The right triceps surae muscle was contracted using a percutaneous electrical stimulation (electrode: Vitrode V Disposable Electrodes, stimulator: SEN-7203, isolator: SS-104J, NIHON KODEN, Tokyo, Japan), while the non-contracted left leg served as control. Muscle contraction was performed for 5 sets of 3-sec stimulations × 10 contractions, with a 7-sec interval between contractions per set, and 3-min rest intervals. After the final set, the rats were euthanized at 3 h and 12 h after the exercise. Among the rats that were euthanized 12 h after exercise, refeeding was performed after exercise for 8 h prior to euthanasia to prevent the influence of fasting.

### RNA Purification

The frozen powdered muscle sample was weighed, and subsequently, total RNA was extracted using a spin column DNA and RNA purification kit (NucleoSpin^®^ TriPrep; Macherey-Nagel, Düren, Germany) following the manufacturer’s instructions. Nucleic acid concentration per milligram of muscle was determined using a spectrophotometer (Nanodrop 2000; Thermo Fisher Scientific, Waltham, MA, United States).

### Ribosome RNA Content Analysis

Ribosomal RNA (28S + 18S rRNA) content analysis was modified and performed as previously reported (Nakada et al., 2016; Ogasawara et al., 2016b). Briefly, 7.5 μl of RNA solution (200 μg tissue/μl) was mixed with × 2 RNA loading buffer (Wako, Osaka, Japan), heated at 70°C for 10 min and immediately chilled on ice. Subsequently, RNA was subjected to electrophoresis using a fluorescence dye (RedSafe^TM^ Nucleic Acid Staining Solution; iNtRON Biotechnology, Gyeonggi-do, South Korea) containing 1% agarose gel in Tris/borate/EDTA (TBE) buffer. The band of 28S + 18S rRNA was filmed using LAS4000 (GE Healthcare, Chicago, IL, United States), and the band intensity was measured using ImageJ software (NIH, Bethesda, MD, United States).

### Western Blot Analysis

The frozen powdered muscle sample was homogenized with radioimmunoprecipitation assay (RIPA) buffer (Cell Signaling Technology, Danvers, MA, United States), protease (cOmplete^TM^, Mini Protease Inhibitor Cocktail; Roche, Basel, Switzerland), and phosphatase inhibitor (PhosSTOP^TM^; Roche, Basel, Switzerland) cocktail. The homogenates were centrifuged at 10,000 × *g* for 10 min at 4°C. The supernatant was collected, and protein concentration was measured using a protein assay rapid kit, following the manufacturer’s instructions (Protein Assay Rapid Kit wako II; FUJIFILM Wako Pure Chemical Corporation, Osaka, Japan). The sample was mixed with Laemmli sample buffer and boiled at 95°C for 5 min. The same amount of sample (20 μg of protein; phosphor-p70S6K was detected using 50 μg of protein) was separated by SDS-PAGE (10 and 15% gels were used). After electrophoresis, the gel was transferred to a polyvinylidene difluoride (PVDF) membrane (Immobilon-P; Merck Millipore, Billerica, MA, United States). Following this, the membrane was blocked with 5% dry skim milk in TBST for 60 min at 22–24°C. The membrane was washed with TBST and incubated with the primary antibody overnight at 4°C. Antibodies against phospho-p70S6K^Thr389^(cat#9234), p70S6K(cat#9202), phospho-4E-BP1^Thr37/46^(cat#9459), 4E-BP1(cat#9452), phospho-ribosomal protein S6^Ser240/244^(cat#2215), ribosomal protein S6(cat#2217), phospho-GSK3β^Ser9^(cat#9336), GSK3β(cat#9315), phospho-eEF2^Thr56^(cat#2331) and total-eEF2 (cat#2332) were purchased from Cell Signaling Technology (Danvers, MA, United States). After overnight incubation, the membrane was washed for 5 min, 3 times, and then incubated with a secondary antibody for 60 min at room temperature. The membrane was treated with chemiluminescent reagents (Luminata Forte Western HRP substrate; Merck Millipore, Billerica, MA, United States), and the bands were filmed using LAS4000 (GE Healthcare, Illinois, CHI, United States); the band intensity was measured using ImageJ (NIH, Bethesda, MD, United States). After chemiluminescent detection, the membrane was stained with Coomassie Brilliant Blue (CBB). All measured protein was normalized by total protein (CBB stained protein ladder).

### Analysis of Muscle Protein Synthesis

Muscle Protein Synthesis was measured using the *in vivo* SUnSET method (Schmidt et al., 2009; Goodman et al., 2011b). Under isoflurane anesthesia, 0.04 μmol puromycin/g body weight (Wako, Tokyo, Japan) diluted in a 0.02 M phosphate-buffered saline (PBS) stock solution was injected intraperitoneally, and the gastrocnemius muscle was removed exactly 15 min after puromycin administration. After the homogenization and centrifugation at 2,000 *g* for 3 min at 4°C, the supernatant was collected and processed for western blotting. Puromycin labeling of the nascent polypeptide was detected using the mouse monoclonal anti-puromycin antibody (cat#MABE343, Merck Millipore, Billerica, MA, United States), which evaluated all protein ladder bands on the membrane.

### Single Myofiber Isolation and Morphometric Analysis

Single skeletal muscle fiber isolation was performed as previously described (Allen et al., 1995). This method is able to remove basal lamina adhering to mononucleated non-muscle and satellite cells (McCall et al., 1998). Briefly, the frozen muscle was thawed at −4°C overnight in glycerol contained 100% relaxing solution. After thawing, single skeletal muscle fibers were dissected using a micro forceps under a stereoscopic microscope (total 100 fibers were isolated from *n* = 5 in each group). The isolated fibers were fixed with 4% paraformaldehyde (PFA) in PBS for 10 min. After the fixation, fibers were placed on MAS-coated slides (Matsunami glass, Osaka, Japan) and mount with 4’,6-diamidino-2-phenylindole (DAPI; Vector Laboratories, Burlingame, CA, United States). Fiber length, sarcomere length, and myonuclear number were counted using a fluorescence microscope (BZ9000; KEYENCE, Osaka, Japan). The myonuclear number per mm fiber was multiplied by the average sarcomere length and divided by 2.5 (resting sarcomere length) to standardize differences in the conditions of stretch. The changes in myonuclear number after HS in gastrocnemius muscle was expressed in Supplementary Figure S6.

### Statistical Analysis

Student *t*-test was applied to compare the change in muscle wet weight after HS. Two-way ANOVA (HS × RE or HS × RT) was applied to evaluate any change in body weight, muscle wet weight, phosphorylated protein, protein, total RNA, rRNA, and protein synthesis using by JMP 10.0 (SAS, Cary, NC, United States). *Post hoc* analyses were performed using *t*-tests, and Benjamini and Hochberg false discovery rate correction was used for multiple comparisons when significant main effects or interactions were confirmed. All values were expressed as mean ± standard error (SE). The level of significance was set at *p* < 0.05.

## Results

### Alteration in Body Weight and Skeletal Muscle Mass After 14-Days of Hindlimb Suspension and Chronic RT

HS group showed significantly lower body weight as compared with CON group after HS and RT (Table 1).

**TABLE 1:** Alteration in body weight after 14-days of hindlimb suspension and chronic RT.

Body weight, g	Pre HS	Post HS	Post RT
CON	361.8 ± 3.9	406.3 ± 1.8*	485.7 ± 8.5*^#^
HS	357.9 ± 2.3	372.1 ± 2.6*^†^	449.5 ± 7.3*^#†^

*p < 0.05 vs. Pre HS, ^#^p < 0.05 vs. Post HS, and ^†^p < 0.05 vs. CON group.

**FIGURE 2:**
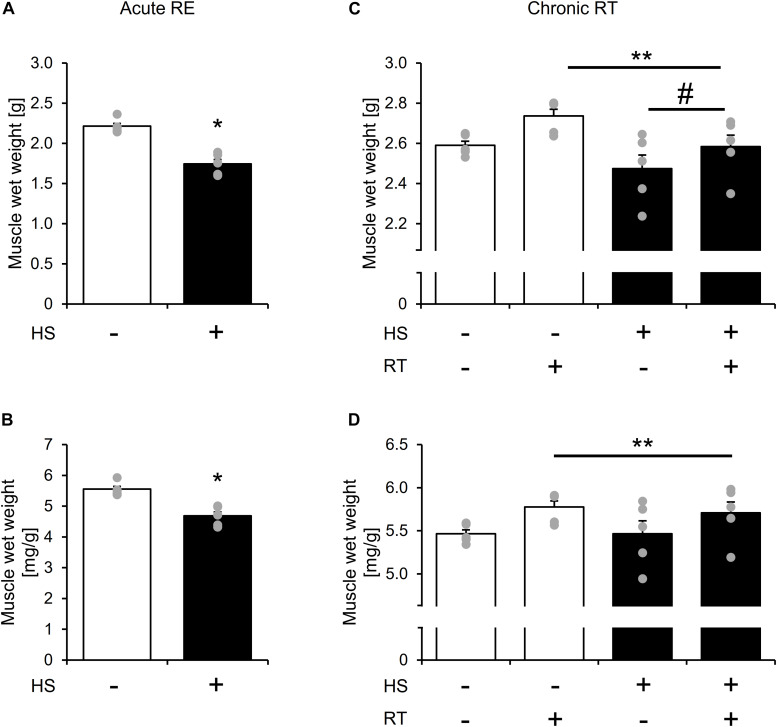
The effect of 14-days of hindlimb suspension and 10-bouts of RT on gastrocnemius muscle mass per body weight. Gastrocnemius muscle wet weight after 14-days of hindlimb suspension **(A,B)**. Gastrocnemius muscle wet weight after chronic RT **(C,D)**. HS: hindlimb suspension. RT: resistance training. Values are means ± SE (*n* = 5/group). *: *p* < 0.05 vs. CON. Black line with **: main effect of RT (*p* < 0.05). Black line with ^#^: main effect of HS (*p* < 0.05).

Changes in gastrocnemius muscle weight after 14-days of HS and chronic RT are shown in Figure 2. Gastrocnemius muscle wet weight was significantly lower in the HS group than in the CON group after 14-days of HS (Figures 2A,B, *p* < 0.05).

Gastrocnemius muscle wet weight remained lower in the HS group after chronic RT (Figure 2C, main effect of HS, and *p* < 0.05). Chronic RT significantly increased gastrocnemius muscle mass in both groups (Figures 2C,D, main effect of RE, and *p* < 0.05). Response of change in muscle mass to contralateral leg was not statistically different between the groups. Gastrocnemius muscle weight is significantly lowere Notably, in the HS group, gastrocnemius muscle mass in the contralateral leg was not statistically different between contralateral leg of the CON group after chronic RT. Data in soleus muscle is shown in Supplementary Figure S1.

### Effect of Acute RE on Muscle Protein Synthesis After HS

**FIGURE 3:**
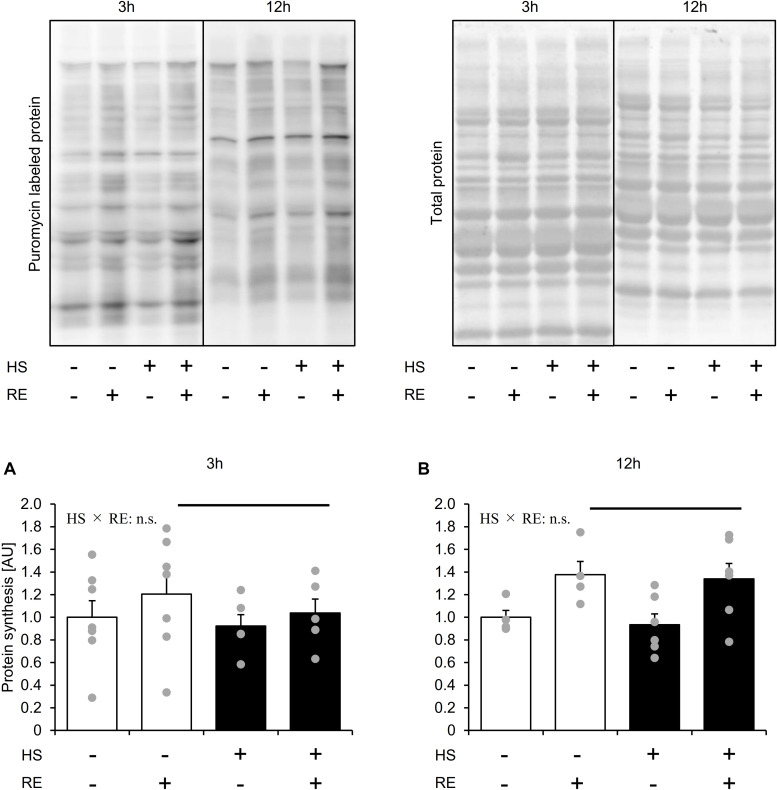
The effect of acute RE on muscle protein synthesis after 14 days of hindlimb suspension **(A,B)**. HS: hindlimb suspension. RE: acute resistance exercise. 3 h: 3-h after exercise. 12 h: 12-h after exercise. Values are means ± SE (*n* = 5–6/group). Black line: main effect of RE (*p* < 0.05).

Statistical differences in MPS were not observed between the control legs of rats in the HS and CON groups (Figure 3). MPS was significantly increased in the exercised leg than in the control leg at 3 h and 12 h after exercise in both the HS and CON groups (Figure 3, main effect of RE, and *p* < 0.05). No significant difference was observed in the exercised legs between the CON and HS groups at either time points (Figure 3). Data for soleus muscle is shown in Supplementary Figure S2.

### Effect of Acute RE on mTORC1 Signaling in Skeletal Muscles After HS

**FIGURE 4:**
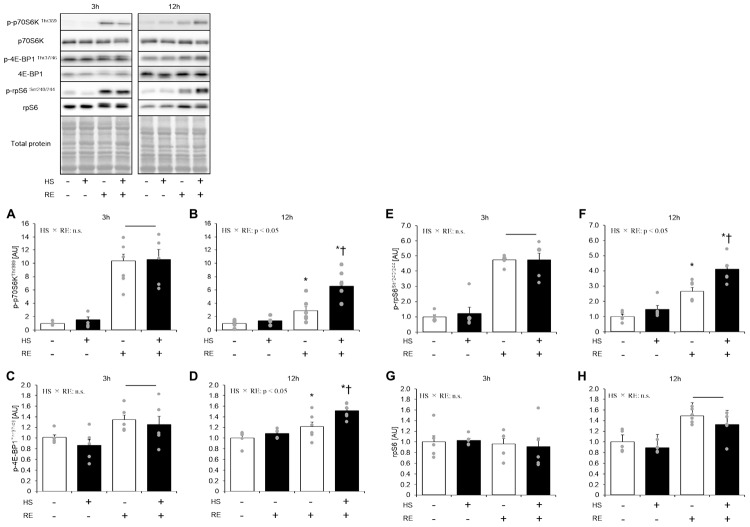
The effect of an acute RE on mTORC1 targeting substrates after 14 days of hindlimb suspension. Changes in the phosphorylation of p70S6K Thr389 in response to acute resistance exercise after hindlimb suspension **(A,B)**. Change in the phosphorylation of 4E-BP1 Thr37/46 in response to acute resistance exercise after hindlimb suspension **(C,D)**. Changes in the phosphorylation of ribosomal protein S6 (rpS6) Ser240/244 in response to acute resistance exercise after hindlimb suspension **(E,F)**. Changes in the expression of ribosomal protein S6 (rpS6) in response to acute resistance exercise after hindlimb suspension **(G,H)**. All changes in protein expression were normalized to total protein. HS: hindlimb suspension. RE: acute resistance exercise. 3 h: 3-h after exercise. 12 h: 12-h after exercise. Values are means ± SE (*n* = 5–6/group). Black line: main effect of RE (*p* < 0.05). *: *p* < 0.05 vs. non-exercised control leg within each group. ^†^: *p* < 0.05 vs. CON group within same condition.

Statistical difference in Thr389 phosphorylation of p70S6K was not observed between the control legs of rats in the HS and CON groups (Figures 4A,B). Phosphorylation of p70S6K on Thr389 was significantly increased at 3 h and 12 h after exercise in both HS and CON groups (Figures 4A,B, *p* < 0.05). Moreover, the magnitude of phosphorylation was significantly higher in the HS group than in the CON group at 12 h after exercise, although the response of p70S6K Thr389 phosphorylation was not different between the CON and HS groups at 3 h after exercise.

Statistical difference in Thr37/46 phosphorylation of 4E-BP1 was not observed between the control legs of rats in the HS and CON groups (Figure 4B). The level of 4E-BP1 phosphorylation on Thr37/46 was significantly increased at 3 h and 12 h after exercise in both the HS and CON groups (Figure 4B, *p* < 0.05 vs. control leg). The magnitude of phosphorylation level was significantly higher in the HS group as compared with the CON group at 12 h after exercise (Figure 4B, *p* < 0.05).

Statistical difference in the total ribosomal protein S6 and Ser240/244 phosphorylation of ribosomal protein S6 was not seen between control legs in the HS and CON groups (Figure 4C). Phosphorylation of ribosomal protein S6 was significantly increased at 3 h and 12 h after exercise in both the HS and CON groups (Figure 4C, *p* < 0.05 vs. control leg). The magnitude of phosphorylation was significantly higher in the HS group than in the CON group at 12 h after exercise. Data for soleus muscle is shown in Supplementary Figure S3.

### Effect of Acute RE After HS on mTOR-Independent Molecules That Associated With Protein Synthesis

**FIGURE 5:**
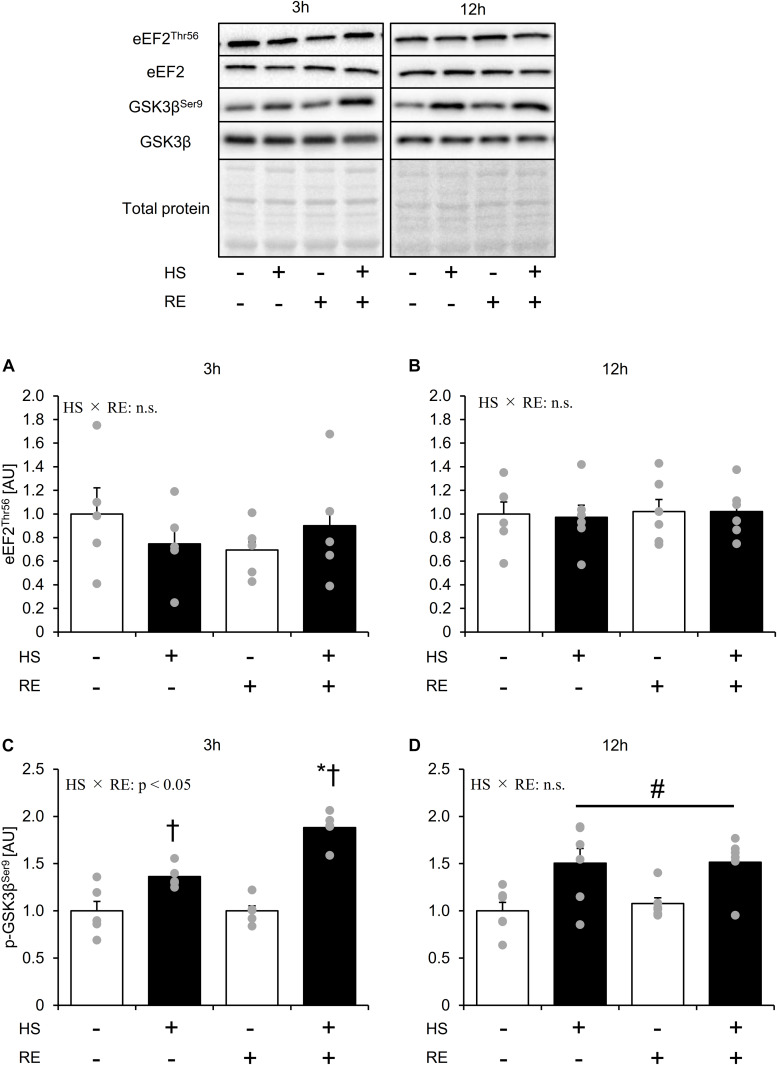
The effect of an acute RE on mTORC-independent substrates related to the protein synthesis after 14 days of hindlimb suspension. Changes in the phosphorylation of eEF2 Thr56 in response to acute resistance exercise after hindlimb suspension **(A,B)**. Changes in the phosphorylation of GSK3β Ser9 in response to acute resistance exercise after hindlimb suspension **(C,D)**. Values are means ± SE (*n* = 5–6/group). Black line with ^#^: main effect of HS (*p* < 0.05). *: *p* < 0.05 vs. non-exercised control leg within each group. ^†^: *p* < 0.05 vs. CON group within the same condition.

Neither phosphorylation at Thr56 nor protein expression of eEF2 was changed by HS and an acute RE (Figure 5A). GSK3β Ser9 phosphorylation was significantly elevated in the HS group (Figure 5B). In addition, acute RE significantly increased GSK3β Ser9 phosphorylation only in the HS group at 3 h after RE. Data for soleus muscle is shown in Supplementary Figure S4.

### Effect of Acute RE on Ribosome RNA Content in Skeletal Muscle After HS

**FIGURE 6:**
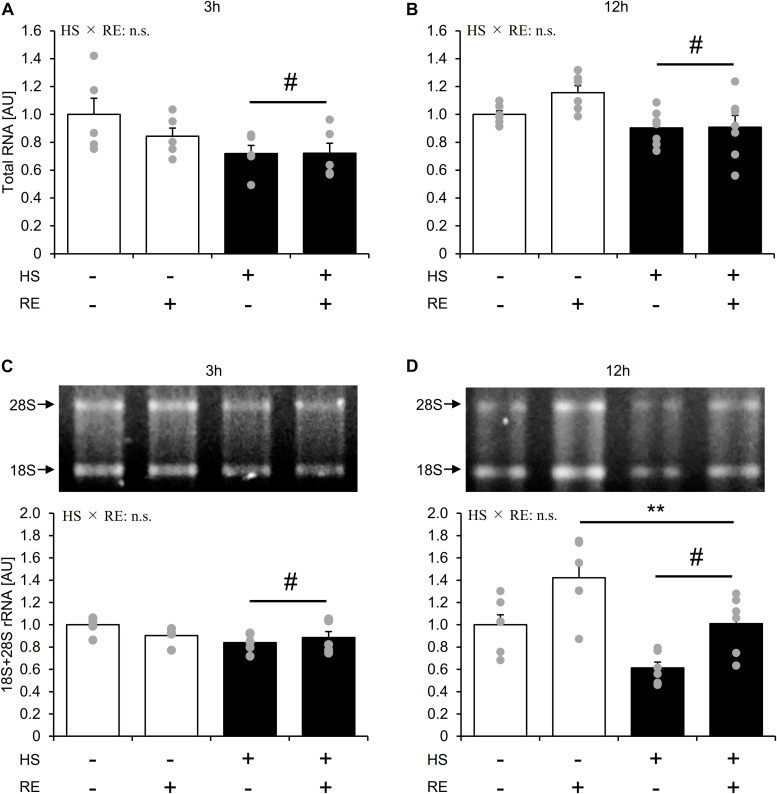
The effect of an acute RE on ribosome RNA content after 14-days of hindlimb suspension. RNA content per mg tissue **(A,B)**. 18S + 28S ribosome RNA **(C,D)**. HS: hindlimb suspension. RE: acute resistance exercise. Values are means ± SE (*n* = 5–6/group). Black line with **: main effect of RE (*p* < 0.05). Black line with ^#^: main effect of HS (*p* < 0.05).

HS group showed significantly lower total RNA content than the CON group at 3 h and 12 h after exercise, respectively (Figure 6A, main effect of HS, and *p* < 0.05). The HS group showed significantly lower 18S + 28S rRNA levels as compared with the CON group at 3 h and 12 h after exercise (Figure 6B, main effect of HS, and *p* < 0.05). At 12 h after exercise, the 18S + 28S rRNA level was significantly increased in the exercised leg compared with the control leg in both groups to the same extent (Figure 6B, main effect of RE, *p* < 0.05). Data for soleus muscle is shown in Supplementary Figure S5.

## Discussion

We investigated whether skeletal muscle disuse atrophy would alter the MPS response to an acute RE and chronic RT-induced muscle mass gain. The main findings of the present study are as follows: (1) skeletal muscle disuse did not alter mTORC1 activity and MPS in the basal state. On the contrary, rRNA content was significantly decreased in atrophied muscles. (2) An acute RE significantly increased MPS and rRNA content, while the response of MPS gain and rRNA increment to RE were not altered after HS. Interestingly, the response of mTORC1 activation to acute RE was higher at 12 h after exercise in atrophied muscles, even though the response of mTORC1 activation was not different at 3 h after exercise. (3) Chronic RT from disuse muscle atrophy significantly increased muscle mass. The response of muscle hypertrophy to RT was not changed after disuse muscle atrophy as compared with RT from basal conditions.

In this study, 14 days of HS significantly decreased gastrocnemius muscle mass, while MPS was unaltered at 14 days after HS, which is a finding consistent with a previous study. A previous study showed that MPS decreases in the early phases of unloading (∼3 days), and then returns to basal levels at 14 days after hindlimb unloading in gastrocnemius muscles (Baehr et al., 2017). Thus, in this study, it was assumed that the level of MPS recovered almost to the basal level. In addition, mTORC1 substrate phosphorylation and expression were also unchanged after 14 days of HS. On the contrary, rRNA content was significantly decreased 14 days after HS. It is generally accepted that protein synthesis is regulated by translational efficiency and translational capacity (Wen et al., 2016). However, in this study, MPS remained unchanged despite the decrease in rRNA, and mTORC1 activity was preserved at 14 days after HS. Although we could not explain this discrepancy, a recent article proposes the possibility that an mTORC1 independent mechanism may modulate basal MPS (Ogasawara and Suginohara, 2018; Ogasawara et al., 2019). GSK3β is reported as a negative regulator of protein synthesis in non-muscle cells (Pal et al., 2017). Further, an *in vitro* skeletal muscle study suggested the potential of GSK3β in the regulation of MPS (Bertsch et al., 2011). In this study, we observed that phosphorylation of GSK3β Ser9, an inhibitory site of kinase activity, was elevated after HS. Thus, GSK3β might contribute to the maintenance of MPS in gastrocnemius muscles during muscle unloading.

In this study, acute RE increased MPS up to 12 h after exercise. In addition, disuse muscle atrophy did not affect the response of MPS to acute RE. Thus, the present results suggest that disuse muscle atrophy does not affect the capacity of MPS to an acute RE. However, interestingly, although mTORC1 activity was significantly higher in HS than that in CON at 12 h after exercise, it did not reflect on the MPS response. Previously, mTORC1 activation has been believed to be as crucial for RE induced MPS response (Burd et al., 2010). However, recent evidence suggests that mTORC1 is contributory, but not necessary to muscle contraction-induced MPS (Ogasawara et al., 2016b; Ogasawara and Suginohara, 2018; You et al., 2018). For these reasons, it is assumed that mTORC1 activity was not necessarily reflected in the MPS response to RE in this study. Moreover, although we could not explain certain causes, mTORC1 activation to RE was sustained in HS. Remarkably, this improved mTORC1 activation to RE was also observed in soleus muscle (Supplementary Figure S3), suggesting that muscle unloading might sensitize mTORC1 activation to an acute RE regardless of the muscle fiber type (e.g., fast twitch or slow twitch dominant). A previous study observed that chronic RT blunted mTORC1 activation response to an acute RE, while a period of detraining re-sensitized mTORC1 activation to an acute RE (Ogasawara et al., 2013). Additionally, You et al. (2015) observed that limb immobilization sensitized MPS response to muscle contraction in a murine model. Thus, chronic contractile activity may modulate the sensitivity of mTORC1 and MPS to muscle contraction. However, recently, Tyganov et al. (2019) have observed that the response of mTORC1 activation and MPS to acute muscle contraction is impaired after muscle unloading in soleus muscle. Although the reason for this discrepancy is unclear, experimental conditions such as muscle contraction (*in vivo* vs. *ex vivo* muscle contraction), unloading period, and sampling time point after muscle contraction may vary the results. Moreover, interestingly, we observed that phosphorylation of GSK3β Ser9 was significantly elevated after an acute RE only in the HS group. Although it was previously observed that GSK3β does not change by RE at basal loaded conditions (Ogasawara and Suginohara, 2018), our observation implicated that GSK3β is specifically involved in muscle regrowth from disuse muscle atrophy. In fact, a previous study observed that skeletal muscle specific knock out of GSK3β facilitates muscle recovery from disuse-induced muscle atrophy (Pansters et al., 2015). A further study should be performed to address the fundamental molecular mechanisms of these phenomena in the regulation of skeletal muscle mass. In this study, since the 3 h group and 12 h group fasted for different times, we did not directly compare their recovery times. Thus, as the limitation, we should be concerned that MPS response and relevant signaling activation might affect by the different fasting times.

Acute RE significantly increased rRNA content as shown in previous reports (West et al., 2016; Ogasawara et al., 2016b). Additionally, the response of rRNA increase was not affected after 14 days of HS even though the rRNA level decreased at the basal state. This implicated that distinct mechanisms regulate rRNA transcription in the basal state and during response to an acute RE. mTORC1 activation is believed to be important for the acute RE-induced rise in rRNA content (West et al., 2016; Ogasawara et al., 2019). However, in the current study, response of rRNA increase to RE was not enhanced even though mTORC1 activation was augmented in HS. Thus, the response of mTORC1 activation to acute RE may not necessarily be reflected by rRNA transcription. Furthermore, even though the rRNA content remained lower after acute RE in the HS group, MPS response was not affected. Thus, the increase in rRNA in response to acute RE may not be related to the MPS response as it has been suggested in a previous study (West et al., 2016). On the other hand, a recent mice study showed that lack of rRNA transcription via p70S6K knock out, impaired the force gain concomitant with muscle hypertrophy to force mTORC1 activation (Marabita et al., 2016). Therefore, it is assumed that ribosome content is important for regulating the skeletal muscle functional integrity during muscle hypertrophy. It was noted that despite the increased rRNA after an acute RE, we could not detect a concomitant change in total RNA expression. Although the mechanism of discrepancy between the change in rRNA content and total RNA content is unclear, one possible explanation is that an difficulty to precisely measure small change in the rRNA, since the total RNA contains other RNAs such as small RNA, tRNA, and mRNA (Figueiredo and McCarthy, 2019).

Finally, we confirmed the response of skeletal muscle hypertrophy to RT from the disuse muscle atrophy state or the basal state. The gastrocnemius muscle mass was significantly increased in both the CON and HS groups by 10-bouts of RT with a similar extent. Thus, results indicated that the response of RT-induced muscle hypertrophy was not modified after disuse muscle atrophy. Previous studies have observed that the skeletal muscle mass gain by reloading from disuse muscle atrophy was greater as compared to that in some of the overload-induced muscle hypertrophies from the basal state using homozoic animals (McCall et al., 1998; Bruusgaard et al., 2012). Gundersen discussed in his review that muscle memory is served as this large response of muscle mass gain from disuse induced muscle atrophy (Gundersen, 2016). However, this comparison is not suitable because the muscle loading pattern is obviously different. In this study, we showed for the first time that the response of RT-induced muscle hypertrophy was not modified after disuse muscle atrophy. Therefore, although the fundamental mechanism of muscle memory remains unclear, it seems that alteration of the responsivity of MPS, and muscle hypertrophy to muscle contraction due to muscle disuse is not relevant. However, it should be noted that the control (reloaded) leg in the HS group, even without any additional exercise sessions, significantly recovered the muscle mass to a similar level as that of the control leg in the CON group during the RT period. In fact, Baehr et al. recently reported that reloading after 14 days of HS was able to recover the gastrocnemius muscle mass similar to the baseline (Baehr et al., 2017). Moreover, the previous study observed that reloading after disuse muscle atrophy robustly increases MPS even in the resting state (Baehr et al., 2016). Therefore, it is likely that reloading itself largely contributes to muscle mass recovery than chronic RT. In addition, it is conceivable that muscle disuse alters the sensitivity of MPS to gravitational loading, but not to high force muscle contraction. Although the detailed mechanism is unclear, previous study suggested that distinct mechanisms regulate skeletal muscle mass recovery and hypertrophy (Chaillou et al., 2015). Further study should address this fundamental mechanism. Also, although we performed the experiment by using 10–12-week-old rats based on previous work (Ogasawara et al., 2016b), MPS and muscle growth is active in young adult than the matured state (Fiorotto et al., 2000). Thus, it is the limitation of this study that perhaps some influence might vary between growing and matured state.

In conclusion, disuse muscle atrophy does not alter the response of MPS to acute RE, despite that the molecular regulations are modified. Furthermore, the response of muscle hypertrophy to chronic RT was not changed after disuse muscle atrophy. Indeed, RT with reloading in the HS group showed larger muscle mass gain from muscle atrophy than the CON group, which performed RT alone as previously suggested (Gundersen, 2016). However, it was noted that a large part of muscle mass recovery was induced by reloading rather than that induced by chronic RT. Therefore, clinical approaches that enable a return to normal life would be a pervasive recovery strategy for muscle mass after prolonged bed rest or immobilization due to surgery or injury.

## Data Availability Statement

All datasets generated for this study are included in the article/Supplementary Material.

## Ethics Statement

The animal study was reviewed and approved by Ethics Committee for Animal Experiments at Ritsumeikan University.

## Author Contributions

SA and SF contributed to the conception and design of the experiments. SA, KK, KS, and SF collected, analyzed, and interpreted the data.

## Conflict of Interest

The authors declare that the research was conducted in the absence of any commercial or financial relationships that could be construed as a potential conflict of interest.
