# Supplementary material for: Response of Resistance Exercise-Induced Muscle Protein Synthesis and Skeletal Muscle Hypertrophy Are Not Enhanced After Disuse Muscle Atrophy in Rat
Source: Front Physiol. 2020 May 21;11:469. doi: 10.3389/fphys.2020.00469 (PMC7258402; doi:10.3389/fphys.2020.00469)
Supplement: Supplementary file 1 [file Data_Sheet_1.docx]

Supplementary Material

#
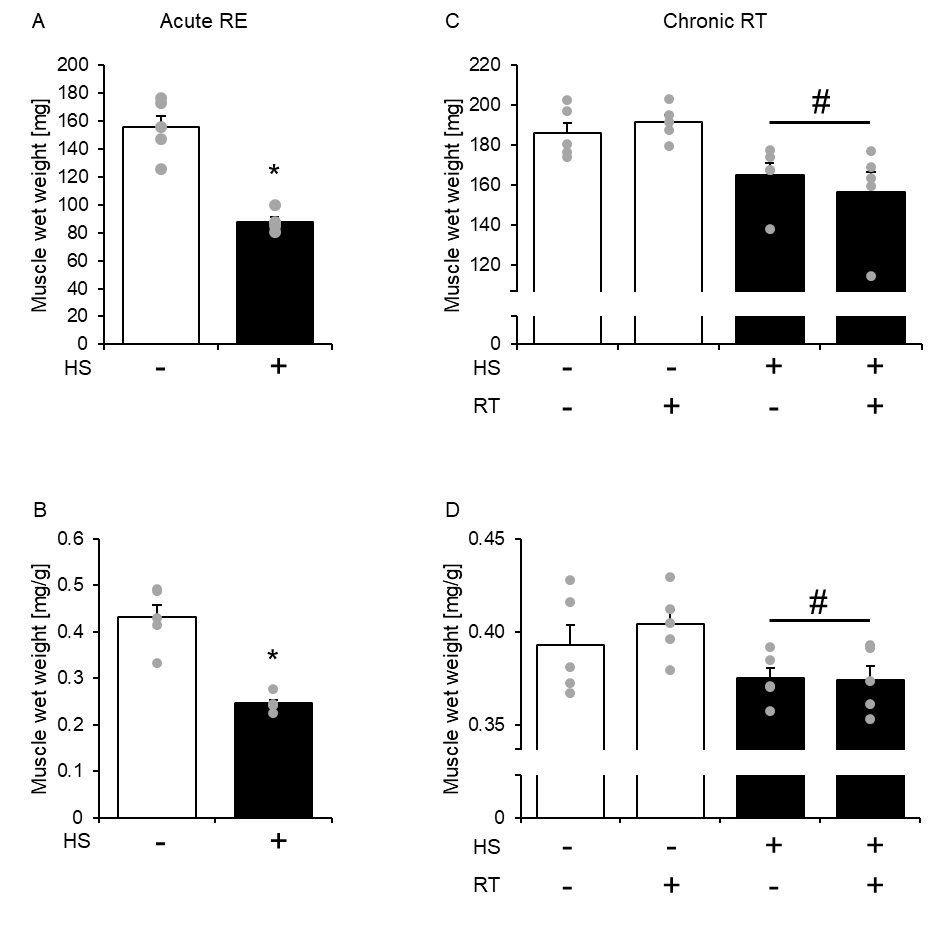


**Supplementary Figure 1.** **The effect of 10-bouts of RT after 14-days of hindlimb suspension on soleus muscle mass**

Soleus muscle wet weight after 14-days of hindlimb suspension (A, B). Soleus muscle wet weight after chronic RT (C, D). HS: hindlimb suspension. RT: resistance training. Values are means ± SE (n=5/group). *: p < 0.05 vs. CON. black line with #: main effect of HS (p < 0.05)


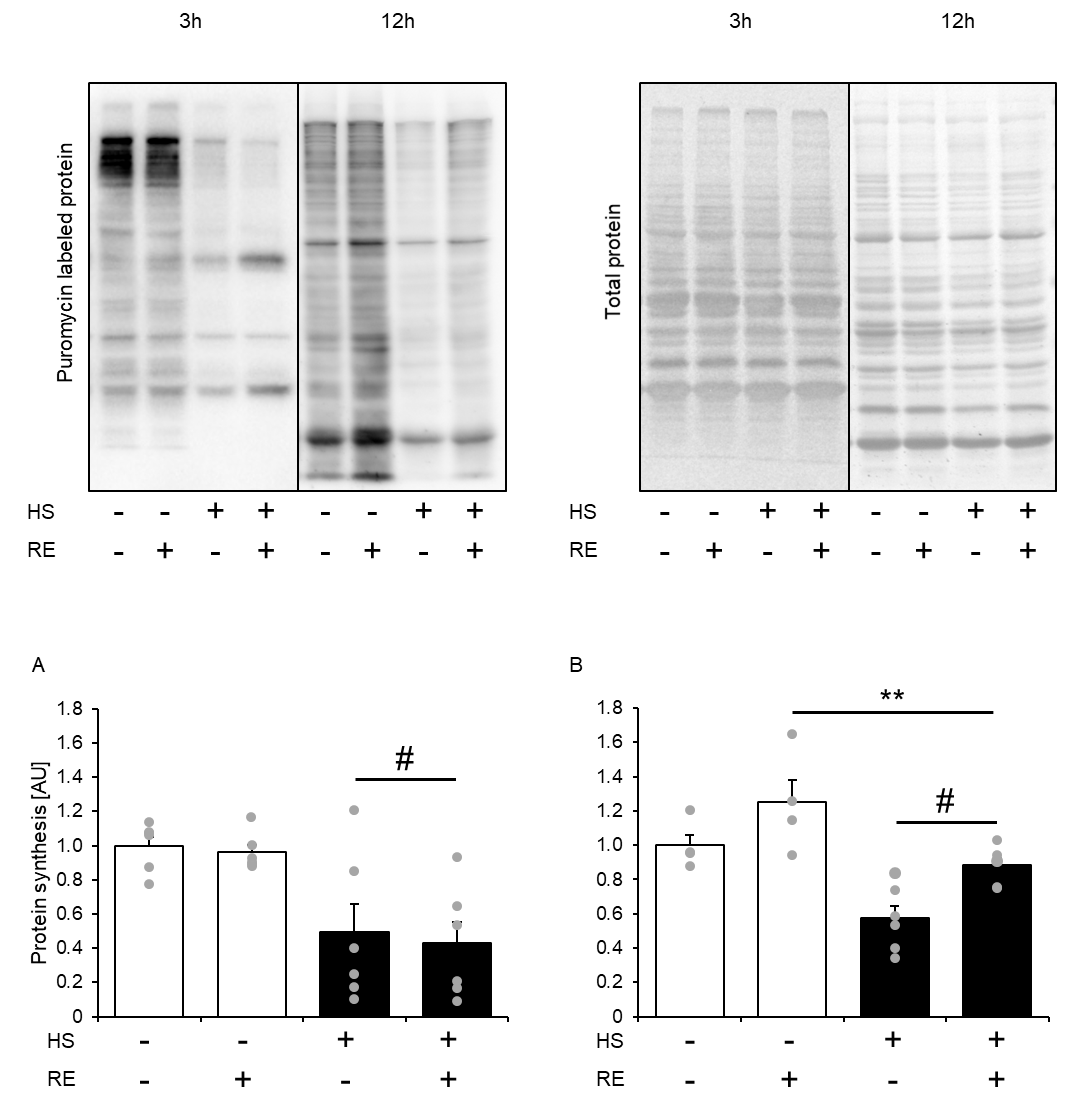


**Supplementary Figure 2. The effect of an acute RE on muscle protein synthesis after 14 days of hindlimb suspension in soleus muscle**

HS: hindlimb suspension. RE: acute resistance exercise. 3h: 3-hour after exercise. 12h: 12-hour after exercise. Values are means ± SE (n=5-6/group). Values are means ± SE. Black line with **: main effect of RE (p < 0.05). Black line with #: main effect of HS (p < 0.05).

**
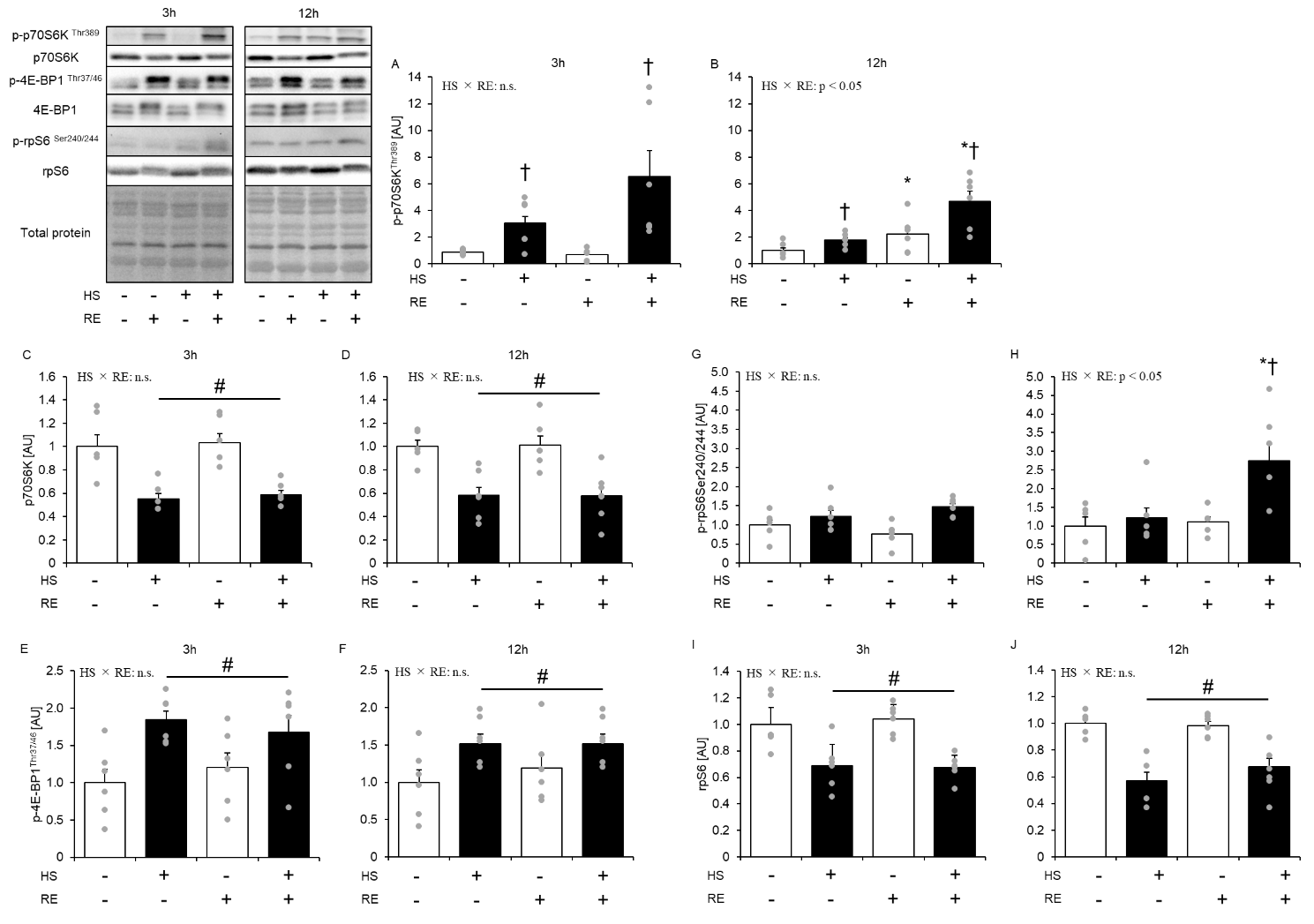
**

**Supplementary Figure 3. The effect of an acute RE on mTORC1 targeting substrates after 14 days of hindlimb suspension in soleus muscle**

Changes in phosphorylation of p70S6K in response to acute resistance exercise after hindlimb suspension (A, B). Changes in total p70S6K in response to acute resistance exercise after hindlimb suspension (C, D). Changes in phosphorylation of 4E-BP1 in response to acute resistance exercise after hindlimb suspension (E, F). Changes in phosphorylation of ribosomal protein S6 (rpS6) in response to acute resistance exercise after hindlimb suspension (G, H). Changes in total ribosomal protein S6 (rpS6) in response to acute resistance exercise after hindlimb suspension (I, J). All changes in protein expression were normalized by total protein. HS: hindlimb suspension. RE: acute resistance exercise. 3h: 3-hour after exercise. 12h: 12-hour after exercise. Values are means ± SE. *: p < 0.05 vs. non-exercised control leg within each group. †: p < 0.05 vs. CON group within same condition. Black line with #: main effect of HS (p < 0.05).

**
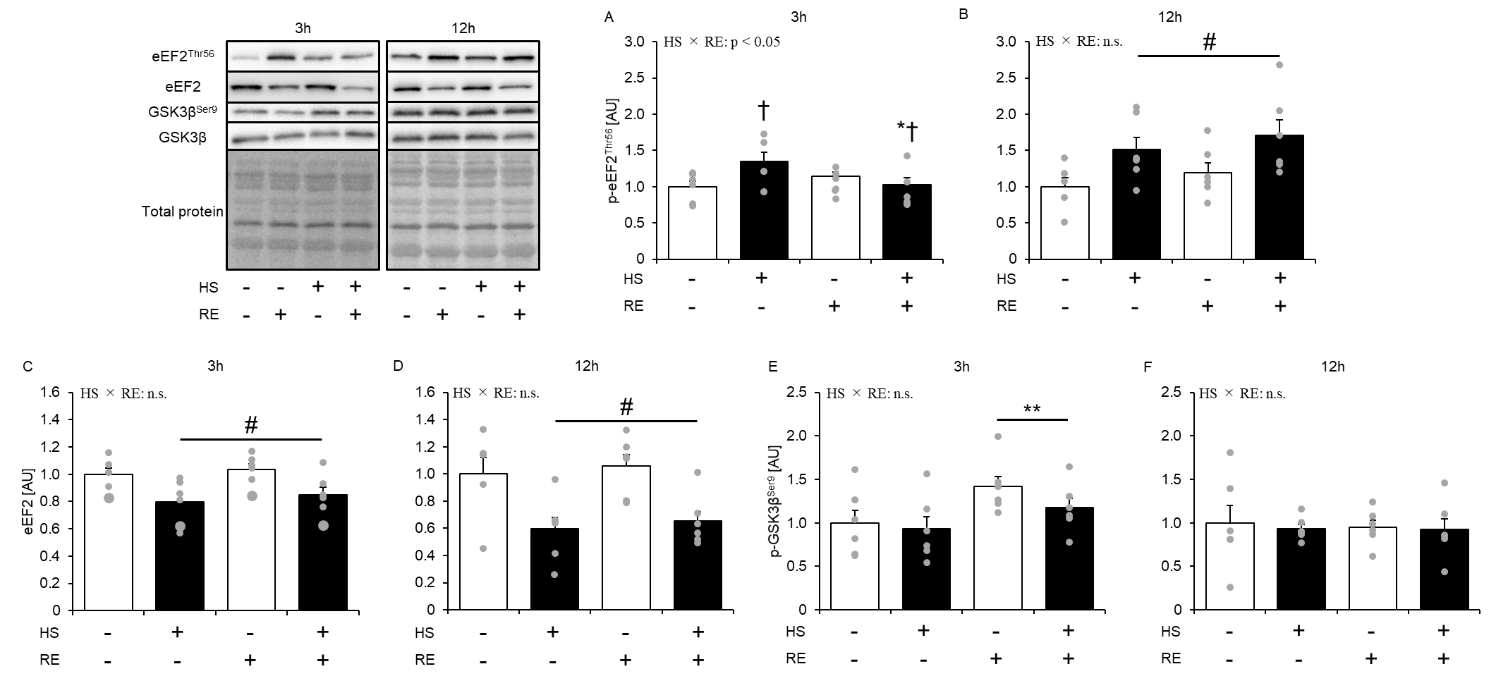
**

**Supplementary Figure 4. The effect of an acute RE after 14 days of hindlimb suspension on mTOR-independent substrates that associate with the protein synthesis in soleus muscle**

Changes in phosphorylation of eEF2 in response to acute resistance exercise after hindlimb suspension (A, B). Changes in total eEF2 expression in response to acute resistance exercise after hindlimb suspension (C, D). Change in phosphorylation of GSK3β in response to acute resistance exercise after hindlimb suspension (B). All changes in protein expression were normalized by total protein. HS: hindlimb suspension. RE: acute resistance exercise. 3h: 3-hour after exercise. 12h: 12-hour after exercise. Values are means ± SE. *: p < 0.05 vs. non-exercised control leg within each group. †: p < 0.05 vs. CON group within same condition. Black line with **: main effect of RE (p < 0.05). Black line with #: main effect of HS (p < 0.05).

**
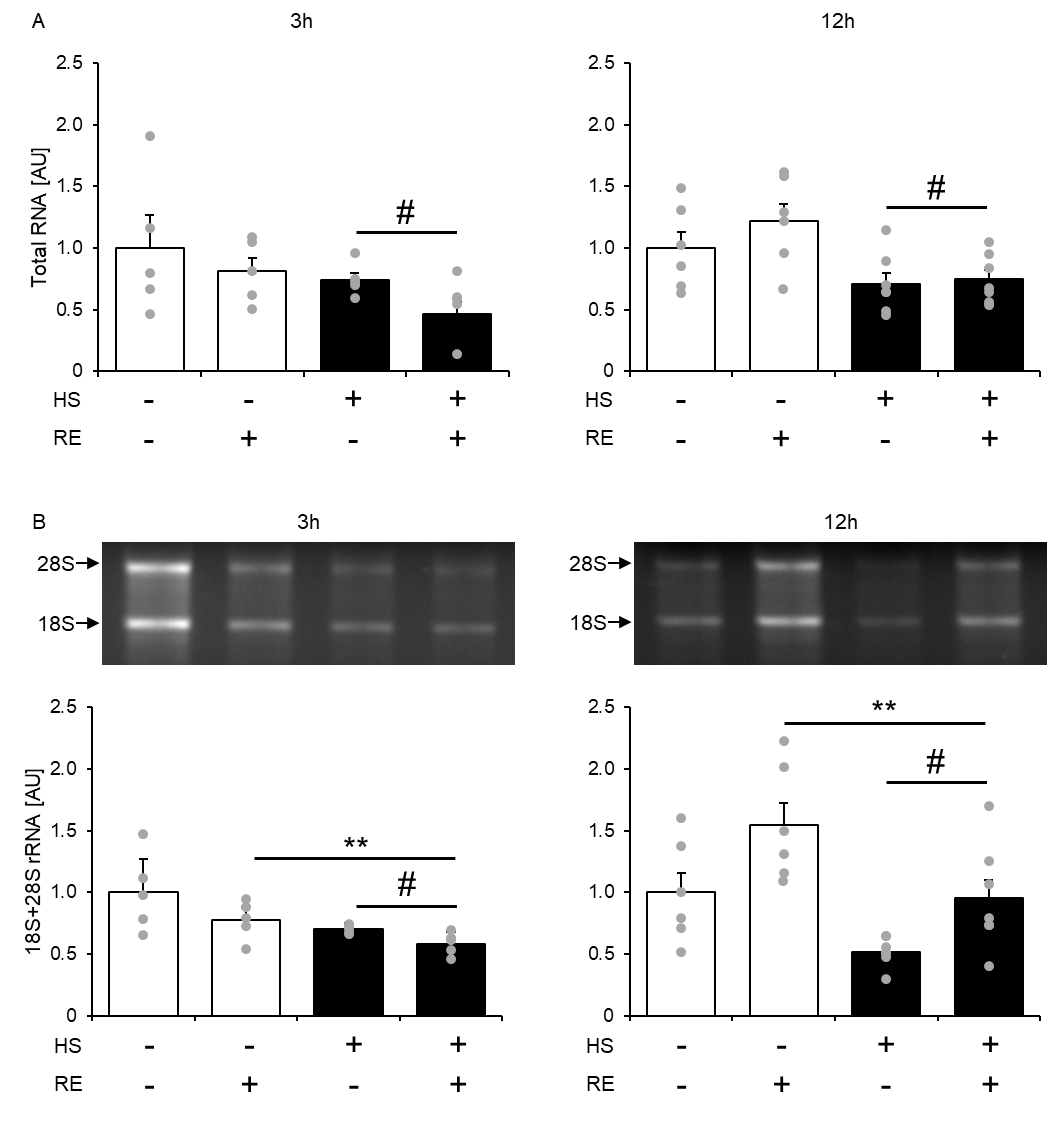
**

**Supplementary Figure 5. The change in ribosome RNA content after 14-days of hindlimb suspension and acute RE in soleus muscle.**

RNA content per mg tissue (A). 18S+28S ribosome RNA (B). CON: control group. HS: hindlimb suspension group. RE: acute resistance exercise. Values are means ± SE. Black line with **: main effect of RE (p < 0.05). Black line with #: main effect of HS (p < 0.05).

**
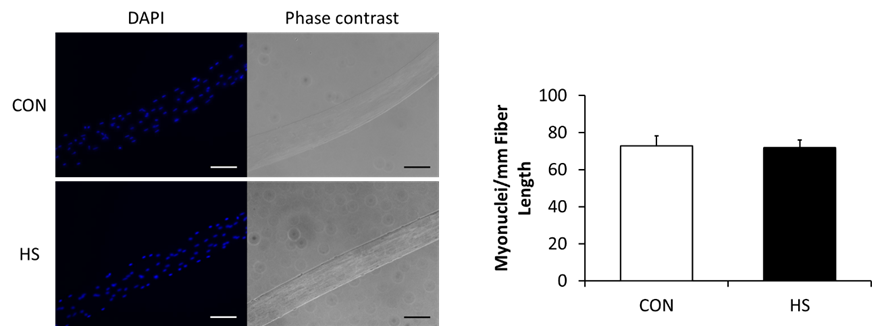
**

**Supplementary Figure 6. Myonuclear number after 14-days of hindlimb suspension in gastrocnemius muscle.**

Micrographs of single gastrocnemius muscle fiber stained with DAPI. Scale bar is 50μm. CON: control group. HS: hindlimb suspension group. Values are means ± SE.
